# Enrichment of extracellular vesicles from tissues of the central nervous system by PROSPR

**DOI:** 10.1186/s13024-016-0108-1

**Published:** 2016-05-23

**Authors:** Xavier Gallart-Palau, Aida Serra, Siu Kwan Sze

**Affiliations:** School of Biological Sciences, Division of Chemical Biology & BioTechnology, Nanyang Technological University, 60 Nanyang Drive, Singapore, 637551 Singapore

**Keywords:** Exosomes, Microvesicles, Human brain, Tissue extraction, Lipidomics, Proteomics, Extracellular vesicles

## Abstract

**Background:**

Extracellular vesicles (EVs) act as key mediators of intercellular communication and are secreted and taken up by all cell types in the central nervous system (CNS). While detailed study of EV-based signaling is likely to significantly advance our understanding of human neurobiology, the technical challenges of isolating EVs from CNS tissues have limited their characterization using ‘omics’ technologies. We therefore developed a new Protein Organic Solvent Precipitation (PROSPR) method that can efficiently isolate the EV repertoire from human biological samples.

**Results:**

In the current report, we present a novel experimental workflow that outlines the process of sample extraction and enrichment of CNS-derived EVs using PROSPR. Subsequent LC-MS/MS-based proteomic profiling of EVs enriched from brain homogenates successfully identified 86 of the top 100 exosomal markers. Proteomic profiling of PROSPR-enriched CNS EVs indicated that > 75 % of the proteins identified matched previously reported exosomal and microvesicle cargoes, while also expanded the known human EV-associated proteome with 685 novel identifications. Similarly, lipidomic characterization of enriched CNS vesicles not only identified previously reported EV-specific lipid families (PS, SM, lysoPC, lysoPE) but also uncovered novel lipid isoforms not previously detected in human EVs. Finally, dedicated flow cytometry of PROSPR-CNS-EVs revealed that ~80 % of total microparticles observed were exosomes ranging in diameter from ≤100 nm to 300 nm.

**Conclusions:**

These data demonstrate that the optimized use of PROSPR represents an easy-to-perform and inexpensive method of enriching EVs from human CNS tissues for detailed characterization by ‘omics’ technologies. We predict that widespread use of the methodology described herein will greatly accelerate the study of EVs biology in neuroscience.

**Electronic supplementary material:**

The online version of this article (doi:10.1186/s13024-016-0108-1) contains supplementary material, which is available to authorized users.

## Background

The emerging roles played by extracellular vesicles (EVs) in cognition, neuropathology and neurotherapeutics have rendered the characterization of these structures from central nervous system (CNS) tissues as a key challenge of current neuroscience [1]. It has been proposed that EVs might mediate the advance of neurodegeneration in amyotrophic lateral sclerosis (ALS) [2], Parkinson’s disease (PD) and Alzheimer’s disease (AD) (see Budnik et al. [3] for detailed review). By contrast, recent reports suggest that EVs may reduce chronic neuroinflammation and may help oligodendrocytes to tolerate oxidative stress insults during neurodegeneration [4–6].

All families of cells in the CNS can release a diverse repertoire of EVs ranging in diameter from 30–1500 nm [7]. These vesicles are released from multivesicular bodies within neurons and glial cells and express hallmark EV proteins including major histocompatibility complex (MHC), endosomal sorting complexes required for transport (ESCRT) and tetraspanins, which have previously been identified as common markers of EVs derived from other tissues [8, 9]. Microvesicles are larger and more heterogeneous than exosomes, and are typically shed from the plasma membrane by randomized blebbing [10]. Due to their increased size, microvesicles may contain fragments of nuclear material or even intact organelles that can be then conveyed to remote sites via the circulation [10]. Although distinctive features based on the diameter range of EVs have been encountered, it is known that exosomes and microvesicles express common markers and share common molecular cargoes what makes imperative their analysis at a global scale [3, 11].

Only trace levels of EVs are present in CNS tissues, hence direct analysis of these bodies and their cargoes by ‘omics’ technologies depends on enrichment strategies that can reduce the background soluble proteins, proteinaceous aggregates, and proteolytic contaminants [12, 13]. While ultracentrifugation is considered the current ‘gold-standard’ method to enrich a wide and untargeted population of EVs from biological samples, this approach is not only time-consuming but also unstandardized and requires large sample volumes [14, 15]. It is in-part due to these technical constraints that, to our knowledge, the CNS-derived EVs repertoire has not yet been characterized in detail, and the potential benefits of exploiting human EVs biology in clinical settings have yet to be determined.

We recently developed a novel method termed PRotein Organic Solvent PRecipitation (PROSPR) that can enrich a diverse repertoire of EVs from human blood plasma, and provides several major advantages over classical ultracentrifugation methods [16]. PROSPR represents a simple, standardized, three-step procedure that pellets soluble proteins and other contaminants during a brief centrifugation cycle while leaving the enriched EVs behind in the organic solvent fraction. In contrast to ultracentrifugation, this quick method is extremely simple to use, enables high-efficient enrichment of EVs from only small amounts of initial sample and additionally the precipitated proteins from hydrophilic tissue can be easily used in further experiments. In the current study, we used an optimized PROSPR protocol to enrich EVs from human and mouse CNS tissues (PROSPR-CNS-EVs), thereby obtaining a diverse arrays of CNS-derived EVs suitable for detailed characterization using ‘omics’ technologies. This new method will significantly aid future studies in the emerging neuroscientific field of EVs biology by simplifying the enrichment of these structures from CNS tissues. Preliminary results were presented in abstract form [17].

## Results

### Lipidomic profile of human PROSPR-CNS- EVs

**Fig. 1:**
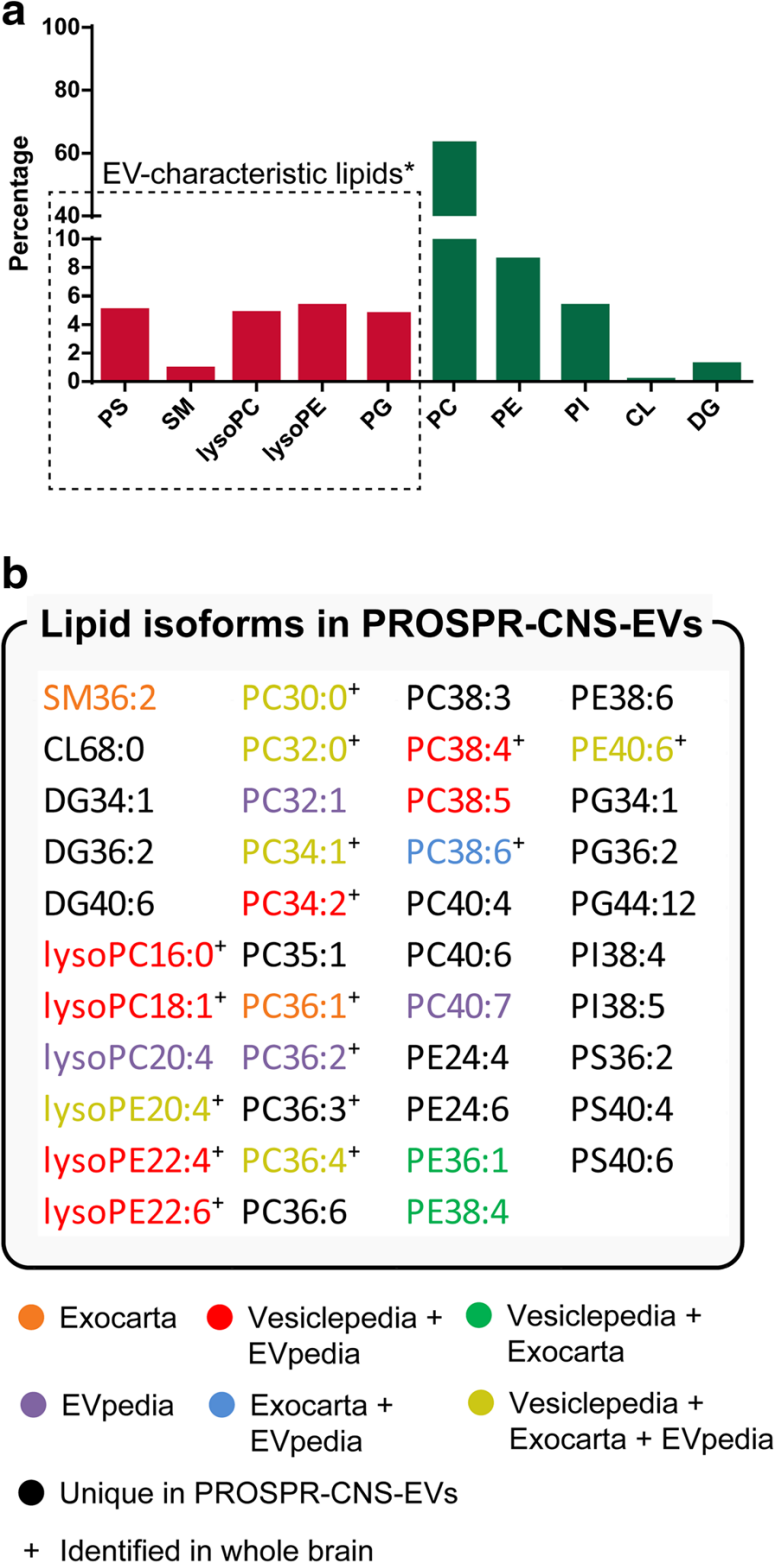
Lipidomics characterization of PROSPR-CNS-EVs. **a** Profile of lipid classes identified in EVs lipidome including percentage of identification of EV-characteristic lipids^*^ [18]. **b** Characterized lipid isoforms were compared with whole brain lipidome (^+^ identifies those lipids found in whole brain lipidome) and matched to records in the EV databases Exocarta [18], Vesiclepedia [19] and EVpedia [21]. Matched lipid isoforms are shown in colors according to their EVs databases match whereas non-matched isoforms are shown in black

Lipidomic profiling of CNS-derived EVs is critical to understand the emerging roles played by these structures in key neurological processes. In the current study, we analyzed the lipidome of PROSPR-CNS-EVs enriched from ~150 mg of human brain using direct infusion electrospray ionization tandem mass spectrometry (ESI-MS/MS) in positive and negative modes (whole list of identifications can be found in Additional file 1: Dataset 1). As shown in Fig. 1a, this approach enabled us to detect 10 different lipid classes, including several hallmark components of human EVs; phosphatidylserines (PS), sphingomyelins (SM), lysophosphatidylcholines (lysoPC), phosphatidylglycerol (PG) and lysophosphatidylethanolamine (lysoPE) [18]. The distribution profile of lipid classes obtained from PROSPR-CNS-EVs was comparable to that obtained from cancer enriched EVs [18].

Further lipidomic characterization of PROSPR-CNS-EVs allowed identification of 43 different isoforms that were searched in the EV databases Exocarta [19, 20], Vesiclepedia [21] and EVpedia [22] (Fig. 1b). This analysis revealed that the majority of lipid isoforms identified in PROSPR-CNS-EVs were novel species that did not match existing EV lipid records (only 37.5 % were registered in Vesiclepedia, 37.5 % in EVpedia and 25.6 % in Exocarta). From these novel EV-associated lipids we highlight the identification of the following isoforms belonging to the EV-characteristic class of lipids PS: PS36:2, PS40:4 and PS40:6. The lipidomic profile of whole brain tissue was also characterized in our study (Additional file 2: Dataset 2). Following this analysis only 16 of the previous 43 lipid isoforms identified in PROSPR fractions were identified in whole brain fractions (Fig. 1b). These results suggested that EVs derived from CNS tissues display characteristic lipid profiles that can be successfully enriched by PROSPR and characterized by lipidomics.

### Proteomic profile of human PROSPR-CNS-EVs

**Fig. 2:**
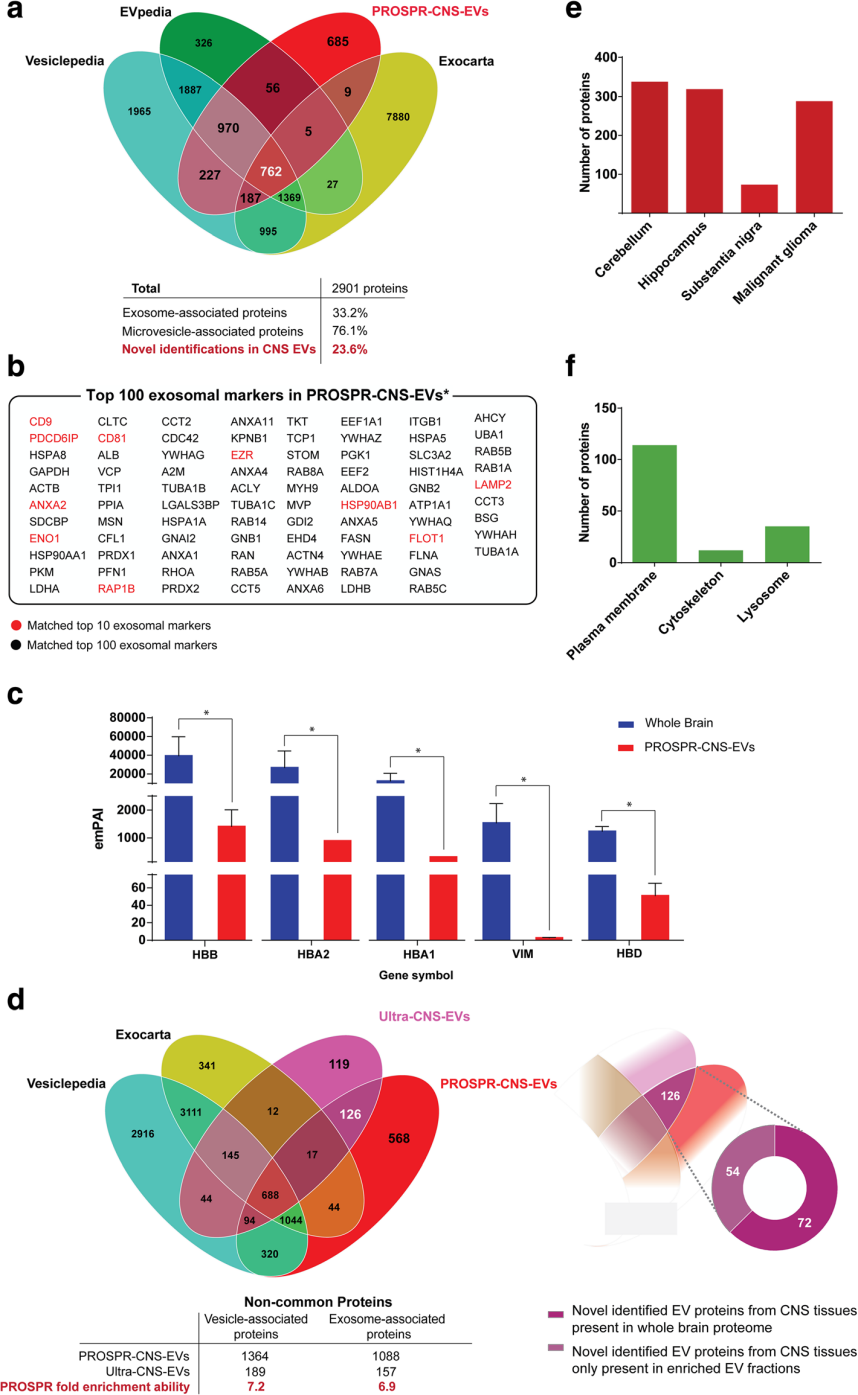
Proteomics characterization of human PROSPR-CNS-EVs. **a** Venn diagram of EV proteins matched to records in the EV databases Exocarta [19], Vesiclepedia [21] and EVpedia [22]. Of the total 2901 proteins identified, 61.8 % were associated with exosome markers and cargoes, 75.4 % were associated with microvesicle markers and cargoes and 23.6 % were novel identifications in brain EVs. **b** List of 86 exosomal markers matched to top 100 exosomal markers in Exocarta. Top 10 of common exosomal markers were indicated in red. **c** Label-free quantitation of the top five most abundant proteins from whole brain proteome compared to their abundance in PROSPR-CNS-EVs. PROSPR-CNS-EV fractions contained on average less than three percent of whole brain amount of the top five highest abundant proteins. **d** Venn diagram of proteins identified from Ultra-CNS-EVs matched to PROSPR-CNS-EVs and EV protein records in Vesiclepedia [21] and Exocarta [19]. Unique 126 EV proteins found in common between Ultra-CNS-EVs and PROSPR-CNS-EVs were compared to whole brain proteome and 72 of these CNS-EV markers were also identified in whole brain whereas 54 were only identified in EVs enriched fractions. Complete list of these proteins is reported in the Additional file 6: Dataset 5 **e** Funrich [24] site of expression analysis of novel identified EV proteins from PROSPR-CNS-EVs fractions. **f** Funrich cellular origin categories of novel identified proteins from PROSPR-CNS-EVs fractions

A total 2901 proteins were identified in PROSPR-CNS-EVs (Additional file 3: Dataset 3). Comparison of these proteins against records in Vesiclepedia and EVpedia [21, 22] revealed that 76.1 % of the total PROSPR-CNS-EV proteome was previously identified in enriched microvesicle fractions (Fig. 2a). Similarly, 33.2 % of the total PROSPR-CNS-EV proteins were identified in enriched exosomal fractions (Fig. 2a), including 86 proteins that matched the top 100 most common exosomal markers listed in Exocarta [19] (Fig. 2b). We achieved high-confidence identification of multiple essential EV markers including Alix, ESCRT proteins, tetraspanins (CD9, CD81 and CD82), and 15 different members of the vacuolar protein sorting family, as well as MHC-I, MHC-II, and the ERM family proteins ezrin, radixin, moesin, flotillin-1 and flotillin-2 (Additional file 3: Dataset 3). We then analyzed the proteome from whole brain considered as the CNS starting material prior to enrichment of EVs (Additional file 4: Dataset 4). We found that only 1228 proteins from whole brain proteome matched previous records in EV-specific databases (Additional file 5: Figure S1a) in front of 2216 proteins that matched same records from PROSPR-CNS-EV fractions (Fig. 2a). Additionally, we quantified by LC-MS/MS label-free quantitation [23] the most abundant proteins in whole brain proteome (HBB, HBA2, HBA1, VIM and HBD). We found that PROSPR fractions contained on average less than three percent of the whole brain amount for these top five high abundant proteins (Fig. 2c). Finally, we analyzed the percentage of membrane proteins in PROSPR-CNS-EVs using FunRich [24], an open software that uses customized pearl scripts to analyze functional and clinical data of proteins compiled in several databases (detailed information of Funrich software can be found in http://funrich.org/faq). Membrane proteins commonly represent ≥ 30 % of the total proteins in EVs-enriched fractions [22, 25, 26] and we found that these proteins represent the 42.43 % of total identified proteins in PROSPR-CNS-EVs. These data further confirmed that PROSPR enables rapid, high-efficient enrichment of EVs from human CNS tissues.

### Comparison of PROSPR and ultracentrifugation techniques for enrichment of CNS-EVs

Data obtained from PROSPR-CNS-EVs was compared to data obtained from sucrose-cushion ultracentrifugation (Ultra-CNS-EVs) as shown in Fig. 2d. In both methodologies EVs were enriched from ~150 mg of human brain tissue. This analysis revealed that PROSPR was able to enlarge the enrichment of vesicle-associated proteins by 7.2-fold and of exosome-associated proteins by 6.9-fold compared to ultra-cushion (Fig. 2d). Additionally, a total of 126 CNS proteins that were not previously identified in EV specialized databases were commonly identified in PROSPR-CNS-EVs and Ultra-CNS-EVs (Fig. 2d). Further analysis of these EV proteins revealed that 72 proteins were also identified in whole brain proteome whereas 54 proteins were only identified in enriched EV fractions (Fig. 2d). Detailed list of these novel CNS-EV molecules can be found in Additional file 6: Dataset 5.

A total of 568 novel species (23.6 % of the total identified proteins) that did not match either with our identified proteins in Ultra-CNS-EVs nor with existing record in EV specialized databases were also identified in PROSPR-CNS-EV fractions (Fig. 2d). These unique proteins from PROSPR-CNS-EV fractions and the 118 unique proteins identified from Ultra-CNS-EVs fractions were then matched with our identified whole brain proteome to determine the percentage of whole brain contamination in enriched EVs. We found that whole brain proteins represented only the 6.9 % of these unique proteins in PROSPR-CNS-EVs compared to the 33.9 % that represented in Ultra-CNS-EVs fractions (Additional file 5: Figure S1b). Furthermore, these novel identified EV molecules from PROSPR fractions included several key brain proteins as multiple S-100 isoforms, brain signaling mediators CAMK2A and sirtuins, neuropeptide NPY, and a wide range of synaptic proteins as synapsins and synaptopodins (a complete list of PROSPR-CNS-EV unique proteins including the 126 common with Ultra-CNS-EV fractions can be found in Additional file 7: Dataset 6).

Functional analysis of these novel PROSPR-CNS-EV identifications was performed in FunRich [24]. This analysis identified a large portion of these proteins as cerebellum and hippocampus characteristic proteins, fact that could provide information about the sites of origin of these novel identified EV-associated molecules in the temporal lobe (Fig. 2e). Furthermore, 15 % of the novel proteins were associated with the cellular plasma membrane, lysosome or cytoskeleton (Fig. 2f). Intriguingly, ~50 % of these previously unknown EV-proteins were also associated with malignant glioma (Fig. 2e), thereby emphasizing the potential of EVs to inform researchers about key pathological processes in the CNS.

### Characterization of human PROSPR-CNS-EVs by dedicated flow cytometry

**Fig. 3:**
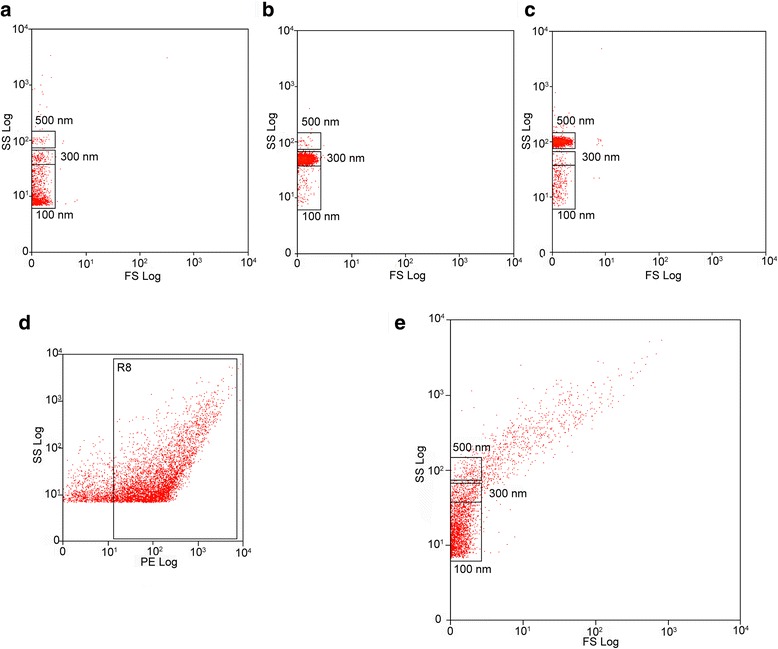
Characterization of PROSPR-CNS-EVs by dedicated flow cytometry (dFC). Microparticle gates for EV size distribution analysis were set after the testing of commercial latex beads from three standard EV sizes. **a** Gate determination of 100 nm beads. **b** Gate determination of 300 nm beads. **c** Gate determination of 500 nm beads. **d** dFC measurement of PE signal from phospholipid-specific stained EVs. The gate for phospholipid-stained vesicles (R8) shows that 83.32 ± 0.65 % of the total material in PROSPR-CNS-EVs was positively stained. **e** Size distribution of EVs by gated analysis of stained particles from PROSPR-CNS-EVs. Lower exosomal range (≤ 100 nm) represented 74.65 ± 2.68 % of total identified microparticles. Higher exosomal range (≤ 300 nm) represented the 8.51 ± 1.11 % of total identified microparticles. Lower microvesicle range (≤ 500 nm) represented the 3.49 ± 0.17 % of total identified microparticles. Higher microvesicle range (> 500 nm) represented the 13.34 ± 1.43 % of total identified microparticles

Size distribution of EVs freshly isolated from human brain tissue was analyzed by dedicated flow cytometry (dFC) as previously described [27, 28]. Microparticle gates were set after the assessment of commercial latex beads from three standard EV sizes (100 nm, 300 nm and 500 nm) (Fig. 3a-c). According to our measurements the EVs stained by the phospholipid-specific dye FM 1-43FX represented the 83.32 ± 0.65 % of the total material in PROSPR-CNS-EV preparations (Fig. 3d). Gated analysis of stained vesicles revealed that 74.65 ± 2.68 % of these particles were placed at the lower exosome range (≤ 100 nm), 8.51 ± 1.11 % of these particles were gated at the higher exosome range (300 nm), 3.49 ± 0.17 % were placed at the lower microvesicle range (500 nm) and the remaining 13.34 ± 1.43 % were revealed as microvesicles displaying diamaters bigger than 500 nm (Fig. 3e). Furthermore, dFC analysis confirmed the low presence of whole organelle contamination in PROSPR-CNS-EVs since less than 10 % of total identified microparticles were detected at the bigger diameter range (≥ 700 nm).

### Proteomic profile of PROSPR-CNS-EVs enriched from mouse tissues

**Fig. 4:**
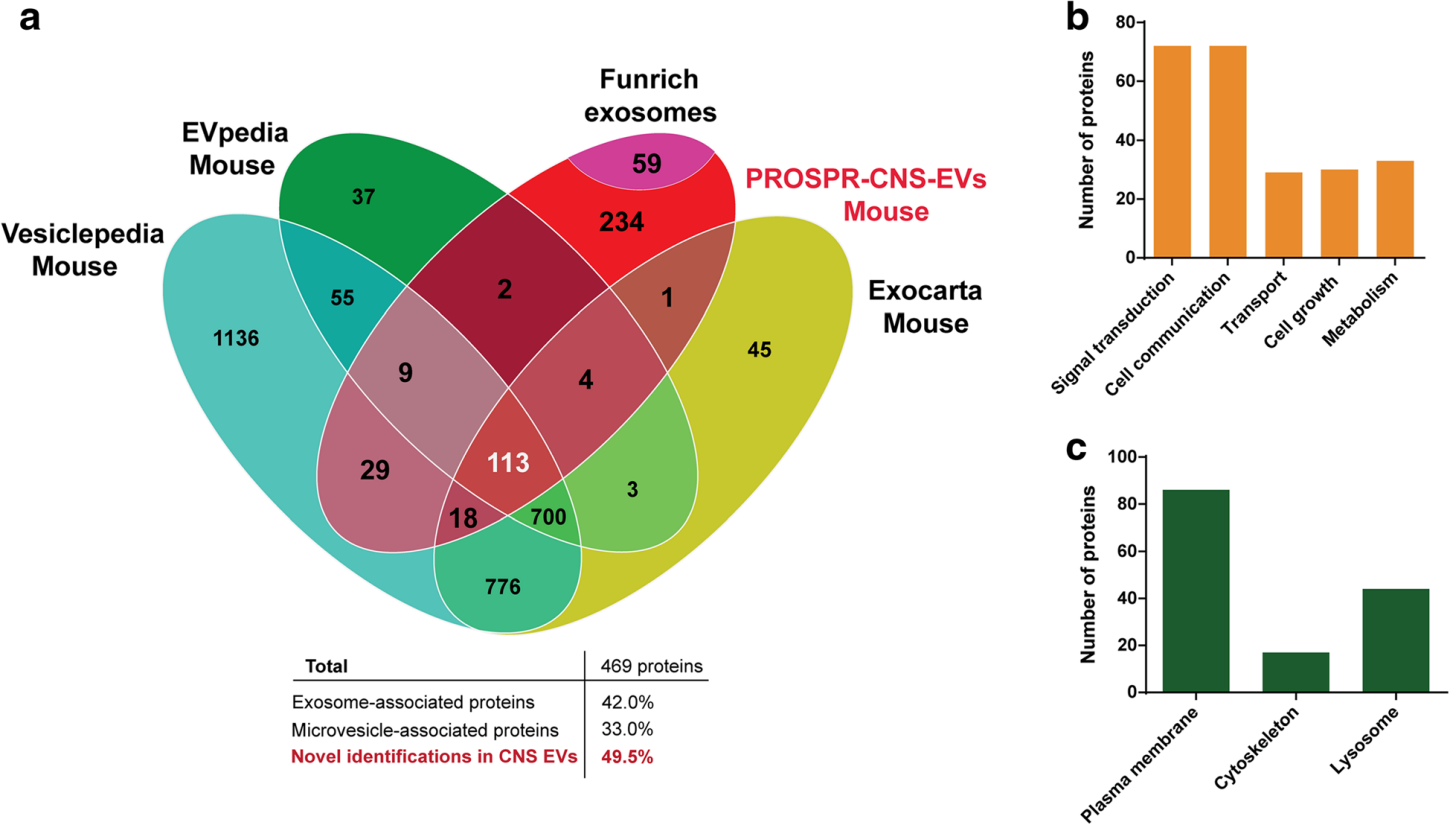
Analysis of PROSPR-CNS-EVs enriched from mouse brain tissue. **a** Venn diagram matched to mouse exosome proteins compiled in Exocarta [19] and Funrich [24] corresponding to the 42 % of total identified proteins in mouse PROSPR-CNS-EV fractions. Mouse microvesicle proteins compiled in Vesiclepedia [21] and EVpedia [22] corresponding to the 33 % of all identified proteins in mouse PROSPR-CNS-EV fractions. Finally, the 49.5 % of the EV proteins found in mouse PROSPR-CNS-EVs were novel identifications not previously found in enriched/isolated EVs. **b** Funrich [24] site of expression analysis of unique EV proteins from mouse PROSPR-CNS-EVs. **c** Funrich cellular origin categories of unique EV proteins from mouse PROSPR-CNS-EVs

A total of 469 proteins were identified in PROSPR-CNS-EVs enriched from ~40 mg of mouse brain tissue (Additional file 8: Dataset 7). From these proteins, the 42 % were matched with previous exosome data compiled in Exocarta and Funrich databases, the 33 % were matched with previous microvesicle data from Vesiclepedia and EVpedia and the 49.5 % were considered novel identifications (Fig. 4a). We then performed functional enrichment analysis of these novel mouse PROSPR-CNS-EV protein identifications as shown in Fig. 4b and c. Signal transduction and cell communication were the most common functions of these analyzed proteins although at a lesser proportion they were also involved on cell growth, cellular transport and metabolism (Fig. 4b). Analysis of cellular association revealed the presence of plasma membrane, cytoskeletal and lysosomal proteins as previously observed in novel EV proteins from human CNS tissues (Fig. 4c). Globally, these results demonstrated that PROSPR was also able to enrich EVs from mouse CNS tissues.

## Discussion

Analysis of EVs released *in vitro* by neuron cells has suggested key roles for these structures on human neurobiology and neuropathology, but extrapolation of these findings to *in vivo* experiments has been hindered by the lack of efficient methods for enriching EVs from CNS tissues. Ultracentrifugation techniques are commonly used to enrich EVs from biological samples, but this approach pellets-down a high amount of contaminating soluble proteins and aggregates that complicate analysis using ‘omics’ platforms [29, 30]. Indeed, while repeated ultracentrifugation coupled with density gradient separation can enable targeted validation of specific proteins in brain exosomes using highly sensitive biochemical techniques [31], the sample volumes generated are often insufficient to permit detailed EV characterization using common ‘omics’ approaches [32]. We therefore developed PROSPR as an easy-to-perform and standardized method that can efficiently enrich EVs from complex biological fluids [16]. PROSPR uses the inexpensive and widely available solvent acetone to precipitate out hydrophilic species and leave intact hydrophobic EVs behind in solution [16, 33]. As expected, we found that ultracentrifugation coupled with a sucrose cushion although it was able to enrich EVs from CNS tissues it was pelleting down ~5 times more of whole brain contaminants than PROSPR. Similarly, due to its ability to clean EVs fractions from hydrophilic contaminants, we found that 7.2-fold more of low abundant vesicle-associated proteins and 6.9-fold more of low abundant exosome-associated proteins were identified in PROSPR-CNS-EVs compared to Ultra-CNS-EVs.

Consistent with previous reports, our proteomic data indicated that PROSPR-CNS-EVs displayed common EV hallmarks and cargoes [19, 21, 22], but also incorporated specific brain proteins not previous identified in EVs. These novel EV components included myelin proteins, multiple synaptic proteins, neurotransmitter receptors, and essential brain kinases. These data were consistent with previous reports demonstrating that EVs mediate intercellular communication between glial cells and neurons [34–36]. Additionally, a large proportion of the previously unknown EV proteins were associated with plasma membrane domains, suggesting that some of these molecules may represent brain-specific EV markers, although further research will be required to confirm this hypothesis. As previously reported by Kim et al. [22], Minciacchi et al. [25], Simpson et al. [26], membrane proteins represent ≥30 % of total proteins identified in quality isolated EV preparations. Consistent with this expectation, membrane proteins in PROSPR-CNS-EVs fractions represented ~43 % of all identified proteins. Furthermore, application of PROSPR to mouse CNS tissues as depicted in methods section of this manuscript, demonstrated that PROSPR was also able to successfully enrich EVs from small amounts of CNS tissue enabling characterization of CNS-EVs from in-vivo disease models.

Circulating CNS-EVs isolated from cerebrospinal fluid contain common EV markers previously compiled in EVs specialized databases [37]. Fiandaca and colleagues found that CNS-EVs released to the blood circulation contain the CNS specific protein neural cell adhesion molecule-L1 (CD171) [38]. Our data were consistent with these previous results and a high number of compiled EV proteins from specialized databases were identified in PROSPR-CNS-EV enriched fractions. Likewise, the CNS-specific protein CD171 was identified in both Ultra-CNS-EV and PROSPR-CNS-EV fractions.

dFC was firstly described by van der Pol and colleagues as the analysis of submicrometer particles using a specially calibrated flow cytometer [39]. Recently, a new method workflow of dFC was developed by Pospichalova and colleagues to characterize the size distribution and relative amount of enriched EVs [28]*.* Characterization of PROSPR-CNS-EVs by dFC as described by Pospichalova and colleagues demonstrated that more than 80 % of the total material in EV preparations was positively stained by the phospholipid-specific dye PKH26 what confirmed, as expected, the high presence of vesicles in enriched PROSPR-CNS-EV fractions. Of note, dFC also revealed that 77 % of the total particles analyzed were found at the exosomal range (100 nm) indicating for the first time that these vesicles are significantly more abundant than microvesicles in CNS tissues.

Analysis of EVs from CNS tissues by LC-MS/MS will not only uncover the protein candidates of the EVs proteome involved in neuropathology but will also enable characterization of the degenerative posttranslational modifications (DPMs) occurring in those proteins [40, 41]. DPMs are considered as crucial events of several neurodegenerative proteinopathies [42–46] notwithstanding their presence and roles in CNS-EVs from *in vivo* tissues have yet to be explored.

In a similar vein, lipid molecules are essential components of bilayer membranes and are thus critical to maintaining EV structural integrity, but can also act as effector molecules in several brain signaling pathways [47, 48]. Data from our PROSPR-CNS-EV lipidome analyses were consistent with previous EV profile reported by Rappa and colleagues in immortalized cancer cells [18]. Three of the lipid isoforms identified in PROSPR-CNS-EVs were novel species not previously reported in EV databases, suggesting that these molecules may represent specific markers of CNS-derived EVs. Our study demonstrates that PROSPR-CNS-EVs can be successfully characterized using lipidomic techniques, which may assist the future identification of novel disease biomarkers, therapies and prognostic tests that exploit lipid biology rather than protein targets [49]. Furthermore, lipidomic characterization of PROSPR-CNS-EVs may allow the generation of novel neurotherapeutic materials mimicking the currently unknown lipoic composition of CNS-EVs [50, 51].

## Conclusions

The novel methodological approach reported herein will enable detailed characterization of the emergent roles played by EVs in human neurobiology. We believe that the simple, inexpensive and standardized application of PROSPR to the analysis of CNS tissues will accelerate progress in our understanding of intercellular communication in the CNS in both healthy and neurodegenerative conditions.

## Methods

### Materials

All reagents were purchased from Sigma-Aldrich (St Louis, MO, USA) unless stated otherwise. Protease inhibitor cocktail tablets were obtained from Roche (Basel, Switzerland) and sequencing-grade modified trypsin was obtained from Promega (WI, USA).

### Brain samples

Human brain tissues were generously provided by the Fukushimura Brain Bank (Toyohashi, Aichi, Japan). Informed consent was obtained and all procedures were approved by the ethics committee of the Fukushimura hospital. The use of human materials was conducted in accordance with the declaration of Helsinki. Brain tissue samples were frozen in liquid nitrogen at the time of autopsy and stored at −150 °C. Human temporal lobe was dissected into small pieces of ~150 mg and larger blood vessels and meninges were removed. Dissected brain tissues from a male C57BL/6 J mouse (~40 mg) were also used. Tissues were washed thrice in 1X PBS for 10 min. All experimental procedures were approved by the Institutional Review Board at Nanyang Technological University and were performed according to institutional guidelines.

### PROSPR-based isolation of EVs from human CNS tissues

#### Homogenization of human CNS tissue

**Fig. 5:**
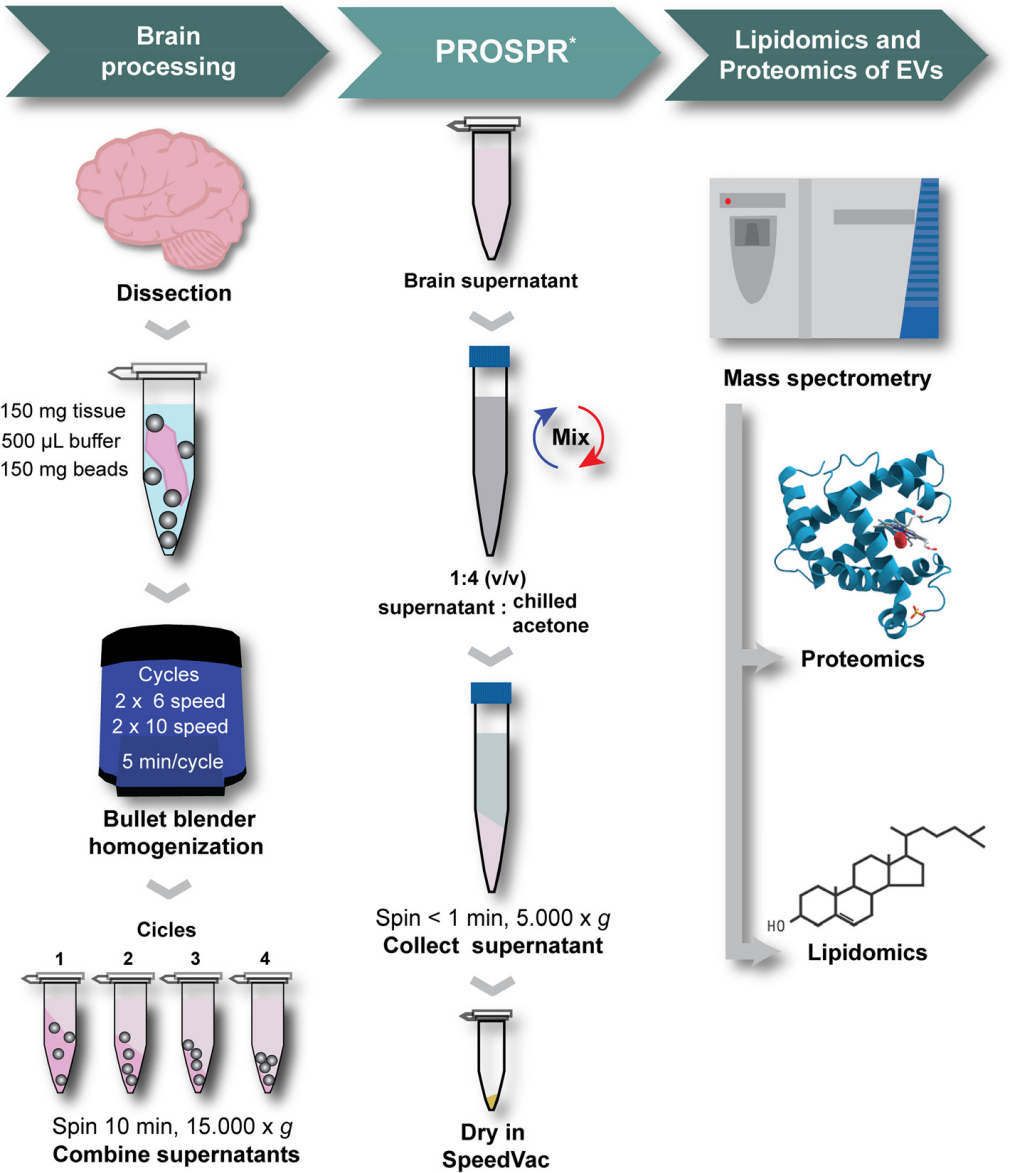
Workflow of the methodology applied to enrich EVs from human CNS tissues. Dissected brain tissue is homogenized in two cycles of medium and higher speed as depicted here. ^*^PROSPR workflow was adapted from our previous report [16]. Obtained PROSPR-CNS-EVs were characterized by lipidomics and proteomics as illustrated in this diagram

Homogenization of human CNS tissue was performed in 100 mM ammonium acetate (AA) buffer supplemented with Complete Protease Inhibitor cocktail using a bullet blender homogenizer (Next Advance, NY, USA) as depicted in Fig. 5. Safe-lock tubes and metallic beads (200 mg of 0.9–2.00 mm particles) were used throughout the homogenization procedure. No denaturing agents were used during this process in order to preserve EV integrity.

CNS tissues were initially homogenized at medium intensity (speed < 6) in two cycles of 5 min each, using 500 μL fresh AA buffer per cycle. The sample was then centrifuged at 15,000 x *g* for 10 min, the supernatant was collected, and the pellet was re-suspended in 500 μL fresh AA buffer for a second round of homogenization for 5 min at high intensity (maximum speed). Next, the sample was centrifuged at 15,000 *x g* for 10 min, the supernatant was collected, and the pellet was subjected to a final round of intense homogenization as described before (after which any remaining pellet was discarded). All homogenization procedure was performed at 4 °C.

#### Homogenization of mouse CNS tissue

Homogenization of mouse CNS tissue was performed as indicated for human CNS tissue although following a milder homogenization procedure. CNS tissue with 500 μL fresh 100 mM AA buffer and 40 mg metallic beads was homogenized at medium intensity (speed < 6) for 5 min. Homogenized sample was centrifuged at 15,000 *x g* for 10 min and the supernatant was collected. Pellet was re-suspended in 500 μL fresh AA buffer and subjected to a second round of homogenization for 5 min performed at speed < 8. Homogenized sample was then centrifuged at 15,000 x g for 10 min and supernatant was collected. All homogenization procedure was performed at 4 °C.

#### PROSPR-based isolation of EVs

All supernatant fractions were then combined and the EVs were enriched by PROSPR as previously described [16]. Briefly, the combined supernatants were mixed with four volumes of chilled acetone (−20 °C) and the mixture was vortexed and centrifuged at 5000 *× g* for less than one minute in order to pellet the precipitated water-soluble proteins and other contaminants. Finally, the PROSPR supernatants containing enriched CNS-derived EVs were collected and dehydrated in a vacuum concentrator (Concentrator Plus, Eppendorf AG, Hamburg, Germany) to obtain the PROSPR-CNS-EVs enriched fraction. The dehydrated pellets were then directly used or stored at −80 °C until further use.

### Ultracentrifugation-based isolation of EVs from human CNS tissues

Isolation of CNS EVs by ultracentrifugation was performed as previously described [16] adapted to CNS tissues. Human brain tissue was homogenized in 100 mM AA buffer as detailed above. Supernatant was then diluted with 1X PBS and transferred to an ultra-centrifugation tube (5 mL) containing 1 mL chilled sucrose (5 % solution). Ultra-centrifugation was done at 200.000 × g, 4 °C overnight to obtain the final Ultra-CNS-EVs enriched pellet.

### Lipidomic characterization of PROSPR-CNS-EVs

Lipidomic characterization of PROSPR-CNS-EVs was performed as previously reported by Milne et al. [52] except for minor modifications. PROSPR-CNS-EVs were re-suspended in a mixture of 800 μL 0.1 N HCl:methanol (1:1, w/w) and 400 μL of chloroform, then vortexed for 1 min, and centrifuged at 18,000 × *g* for 5 min. The chloroform lower-phase containing lipids was then aspirated into a new tube and evaporated in a vacuum concentrator. The lipids were then reconstituted in 150 μL methanol:chloroform (9:1, v/v). Finally, a 1.5 μL volume of ammonium hydroxide was added to the sample to ensure proper protonation for subsequent analysis by mass spectrometry. Lipid characterization by direct infusion ESI-MS/MS was performed using a Thermo Scientific Inc. Orbitrap Elite mass spectrometer (Bremen, Germany). The sample was sprayed at a flow-rate of 1 μL/min using a Thermo Scientific Inc. Ion Max API source.

Data were acquired over 4.5 min/run using LTQ Tune Plus software (Thermo Scientific Inc., Bremen, Germany) in either positive or negative mode. For both modes, data acquisition was performed by alternating between Full FT-MS (150–1100 m/z, resolution 120,000 [at 400 m/z], with one μscan per spectrum) and FT-MS/MS with the 10 most intense ions above a 500 minimum signal threshold being fragmented in collision-induced dissociation mode at 35 % normalized collision energy (with an automatic mass range selection, resolution 120,000 [at 400 m/z] and 1 μscan per spectrum). Dynamic exclusion list was enabled with an exclusion duration of 45 s and an exclusion list size of 30. Capillary temperature was set at 250 °C and source voltage at 1.5 kV. The automatic gain control (AGC) target for FT-MS and MS/MS was set at 1e10^6^.

### Proteomic characterization of PROSPR-CNS-EVs

#### In solution digestion

PROSPR-CNS-EVs were lysed and the constituent proteins were digested as previously described [16] except for minor modifications. Dehydrated PROSPR-CNS-EVs were completely re-dissolved in a 16 M urea, 50 mM ABB buffer, and then diluted 1:1 with bi-distilled water. The proteins were then reduced with 10 mM dithiotreitol at 30 °C for 3 h and subsequently alkylated with 20 mM iodoacetamide for 45 min at room temperature in the dark. Urea was then diluted to < 1 M using 25 mM ABB and the proteins were digested overnight at 30 °C with trypsin at 1:20 protein-to-enzyme ratio (w/w). The reaction was quenched by acidification to a 0.5 % final concentration of formic acid (FA). Peptides were desalted using Waters Sep-pack 50 mg C18 cartridges (Waters, MA. USA) and the eluted peptide solution was dried in a vacuum concentrator (Eppendorf, Hamburg, Germany).

### High-pressure liquid chromatography fractionation

Fractionation of peptides by high-pressure liquid chromatography (HPLC) was performed as previously described in [16] and [53]. Desalted peptides were re-dissolved in 200 μL mobile phase A (85 % acetonitrile (ACN), 0.1 % acetic acid). Fractionation into a Fortis Amino column (4.6 × 200 mm, 3 μm, Fortis Technologies Ltd., Cheshire, UK) was performed using a Shimadzu Prominence UFLC system with wavelength monitoring at 280 nm. Separation of peptides was carried out using a 72-min gradient at a flow-rate of 1 mL/min as follows; 0 % mobile phase B (10 % ACN, 0.1 % FA) for 5 min, 0–20 % mobile phase B for 25 min, 20–33 % mobile phase B for 10 min, 33–60 % mobile phase B for 10 min, and 60–100 % mobile phase B for 5 min, followed by 17 min at 100 % mobile phase B. The collected fractions were then combined into consecutive pairs or according to peak intensities and dried in a vacuum concentrator.

### LC-MS/MS

Peptides were reconstituted in 3 % ACN, 0.1 % FA before being subjected to LC-MS/MS analysis using a Dionex UltiMate 3000 UHPLC system coupled with an Orbitrap Elite mass spectrometer (Thermo Scientific Inc., Bremen, Germany). The sample was sprayed using a Michrom Thermo CaptiveSpray nanoelectrospray ion source (Bruker-Michrom Inc., Auburn, USA) with a source voltage of 1.5 kV. Sample peptides (~2 μg per injection) were separated at a flow rate of 300 nL/min using a reverse-phase Acclaim PepMap RSL column (75 μm ID × 15 cm, 2 μm particle size, Thermo Scientific Inc.) maintained at 35 °C. Separation of peptides was performed using a 60-min gradient starting with 3 % mobile phase B (90 % ACN, 0.1 % FA) and 97 % mobile phase A (0.1 % FA in water) for 1 min, 3–35 % mobile phase B for 47 min, 35–50 % mobile phase B for 4 min. The gradient was then increased to 80 % mobile phase B over 6 s, maintained under isocratic conditions for 78 s, then reverted to initial conditions over 6 s, and again maintained under isocratic conditions for 6.5 min.

Data acquisition in positive mode was performed using LTQ Tune Plus software (Thermo Scientific Inc., Bremen, Germany) alternating between full FT-MS (350–1600 m/z, resolution 60,000 [at 400 m/z], with one μscan per spectrum) and FT-MS/MS (150–2000 m/z, resolution 15,000 [at 400 m/z], one μscan per spectrum). The 10 most intense ions with a threshold of 500 counts were fragmented in high-energy collisional dissociation (HCD) mode using 32 % normalized collision energy. The AGC target for full FT-MS and MS/MS was set to 1e + 06, precursor ion charge state screening was activated, and capillary temperature was set to 250 °C.

### Characterization of PROSPR-CNS-EVs by dFC

PROSPR-CNS-EV fractions were concentrated to ~50 μL using the vacuum concentrator. Fluorescent labeling of PROSPR-CNS-EVs was performed using the phospholipid-specific dye PKH26 according to manufacturer’s instructions (PKH26-GL, Sigma-Aldrich, St Louis, MO, USA). Cyan-ADP flow cytometer (Beckman Coulter, CA., USA) equipped with three solid state lasers (407 nm, 488 nm, and 633 nm) was used for data acquisition. Fluorochrome excitation at 488 nm and acquisition at 585 nm were kept parameters during data acquisition of dyed EVs. Three EVs reference latex beads of 0.1 μM, 0.3 μM and 0.5 μM from Beckman Coulter (CA, USA) were used to set PMT voltages and thresholds for light scattering. Non-labeled PROSPR-CNS-EV samples, bi-distilled water and PBS buffer were used as negative controls. All measurements were performed in log mode with noise levels set to side scatter at 0.08. Microparticles were measured setting the stop condition for PE TruCount particles at 5.000 and 20.000 events. All dFC experiments were performed in triplicate.

### Bioinformatics and data analysis

Raw MS/MS data from the lipidomic and proteomic experiments were de-isotoped and converted into mascot generic format files using Thermo Proteome Discoverer version 1.4.1.14 (Thermo Scientific Inc., Bremen, Germany). Identification of lipids was conducted using in-house software and LipidBlast [54] in high-throughput mode together with NIST MSPepSearch (http://peptide.nist.gov/). Database searches for lipid identification used all available LipidBlast libraries for positive and negative modes. Precursor ion tolerance and fragment peak tolerance were set at 0.01 m/z. Relative quantification of lipid families was based on the absolute number of identifications in a total of 5 replicates from each analyzed mode (Additional file 1: Dataset 1). Database searches for proteomic data were performed using an in-house Mascot server (version 2.3.02, Matrix Science, MA, USA) with a precursor ion tolerance of 10 ppm and fragment peak tolerance of 0.05 Da as previously indicated [55]. Uniprot human database (downloaded on 15^th^ July of 2015, 90478 sequences and 35.890.546 residues) was used for database search. Two missed trypsin cleavage sites were tolerated. Carbamidomethylation (C) was set as fixed modification and deamidation (NQ) and oxidation (M) were set as variable modifications. Peptides with Mascot score >15 were used to generate the peptide list in all tested human conditions. Additionally, Uniprot mouse database (downloaded on 19^th^ February of 2016, 101382 sequences and 43.874.034 residues) was used for database search. The rest of search conditions for mouse PROSPR-CNS-EV fractions were the same already described for human database search.

To filter non-confident peptide and protein identifications in human PROSPR-CNS-EVs, whole brain proteome and Ultra-CNS-EVs; only those proteins identified in at least two replicates were reported. Label-free quantitation of protein abundance was conducted using the exponentially modified protein abundance index (emPAI) in Mascot [23] and functional enrichment analyses were performed in the open software Funrich [24]. Statistical cut-off of enrichment analyses in FunRich software was kept as default with a p-value <0.05 after Bonferroni correction. Venn diagrams were performed using the open software Venny [56].

### Data availability

The lipidomic and proteomic data generated in this study have been made publicly available through the ProteomeXchange Consortium [57] via the partner repository PRIDE. Identifier: PXD003288.

## Additional files

Additional file 1: Dataset 1. List of lipid isoforms identified by direct infusion ESI-MS/MS in PROSPR-CNS-EVs. Database searches for lipid identification used all available LipidBlast libraries for positive and negative modes. Precursor ion tolerance and fragment peak tolerance were set at 0.01 m/z. Absolute number of identifications was generated considering 5 replicates from each analyzed mode. (XLS 31 kb) Additional file 2: Dataset 2. List of lipid isoforms identified by direct infusion ESI-MS/MS in whole brain. List of lipid isoforms identified by direct infusion ESI-MS/MS in PROSPR-CNS-EVs. Database searches for lipid identification used all available LipidBlast libraries for positive and negative modes. Precursor ion tolerance and fragment peak tolerance were set at 0.01 m/z. Absolute number of identifications was generated considering 5 replicates from each analyzed mode. (XLS 28 kb) Additional file 3: Dataset 3. List of proteins identified by LC-MS/MS in PROSPR-CNS-EVs. Database searches was performed using an in-house Mascot server (version 2.3.02, Matrix Science, MA, USA) with a precursor ion tolerance of 10 ppm and fragment peak tolerance of 0.05 Da. Uniprot human database (downloaded on 15th July of 2015, 90478 sequences and 35.890.546 residues) was used for database search. Two missed trypsin cleavage sites were tolerated. Carbamidomethylation (C) was set as fixed modification and deamidation (NQ) and oxidation (M) were set as variable modifications. Peptides with Mascot score >15 were used to generate the peptide list in all tested human conditions. (XLS 543 kb) Additional file 4: Dataset 4. List of proteins identified in human whole brain. Database searches was performed using an in-house Mascot server (version 2.3.02, Matrix Science, MA, USA) with a precursor ion tolerance of 10 ppm and fragment peak tolerance of 0.05 Da. Uniprot human database (downloaded on 15th July of 2015, 90478 sequences and 35.890.546 residues) was used for database search. Two missed trypsin cleavage sites were tolerated. Carbamidomethylation (C) was set as fixed modification and deamidation (NQ) and oxidation (M) were set as variable modifications. Peptides with Mascot score >15 were used to generate the peptide list in all tested human conditions. (XLS 286 kb) Additional file 5: Figure S1. Characterization of whole brain proteome. **a.** Venn diagram analysis of whole brain proteome matched to records in Exocarta [19], Vesiclepedia [21] and EVpedia [22] databases. **b.** Venn diagram analysis of whole brain proteome matched to the 568 unique proteins obtained from the comparison between PROSPR-CNS-EVs versus proteins compiled in Exocarta [19] and Vesiclepedia [21] and to the 119 unique proteins obtained in common with Ultra-CNS-EVs fractions (see in Fig. 2d the origin of these unique CNS-EV proteins). (TIF 1726 kb) Additional file 6: Dataset 5. List of 72 novel identified EV proteins from CNS tissue present in whole brain proteome. List of 54 novel identified EV proteins from CNS tissue only present in EV-enriched fractions. (XLS 322 kb) Additional file 7: Dataset 6. List of proteins identified in PROSPR-CNS-EVs that were not previously reported in EV databases. (XLS 146 kb) Additional file 8: Dataset 7. List of proteins identified in PROSPR-CNS-EVs from mouse brain. Database searches was performed using an in-house Mascot server (version 2.3.02, Matrix Science, MA, USA) with a precursor ion tolerance of 10 ppm and fragment peak tolerance of 0.05 Da. Uniprot mouse database (downloaded on 19th February of 2016, 101382 sequences and 43.874.034 residues) was used for database search. Two missed trypsin cleavage sites were tolerated. Carbamidomethylation (C) was set as fixed modification and deamidation (NQ) and oxidation (M) were set as variable modifications. Peptides with Mascot score >15 were used to generate the peptide list in all tested human conditions. (XLS 127 kb)

## Abbreviations

AA: Ammonium acetate
ABB: Ammonium bicarbonate
ACN: Acetonitrile
AGC: Automatic gain control
CNS: Central nervous system
dFC: Dedicated flow cytometry
DPMs: Degenerative posttranslational modifications
ESCRT: Endosomal sorting complexes required for transport
ESI-MS/MS: Electrospray ionization tandem mass spectrometry
EVs: Extracellular vesicles
FA: Formic acid
HPLC: High-pressure liquid chromatography
LC-MS/MS: liquid chromatography tandem-mass spectrometry
lysoPC: Lysophosphatidylcholines
lysoPE: Lysophosphatidylethanolamine
PROSPR: Protein organic solvent precipitation
PROSPR-CNS-EVs: Extracellular vesicles enriched by PROSPR
PG: Phosphatidylglycerol
PS: Phosphatidylserines
SM: Sphingomyelins
Ultra-CNS-EVs: Extracellular vesicles enriched by ultracentrifugation
