# Supplementary figures and images for: Enrichment of extracellular vesicles from tissues of the central nervous system by PROSPR
Source: Mol Neurodegener. 2016 May 23;11:41. doi: 10.1186/s13024-016-0108-1 (PMC4877958; doi:10.1186/s13024-016-0108-1)

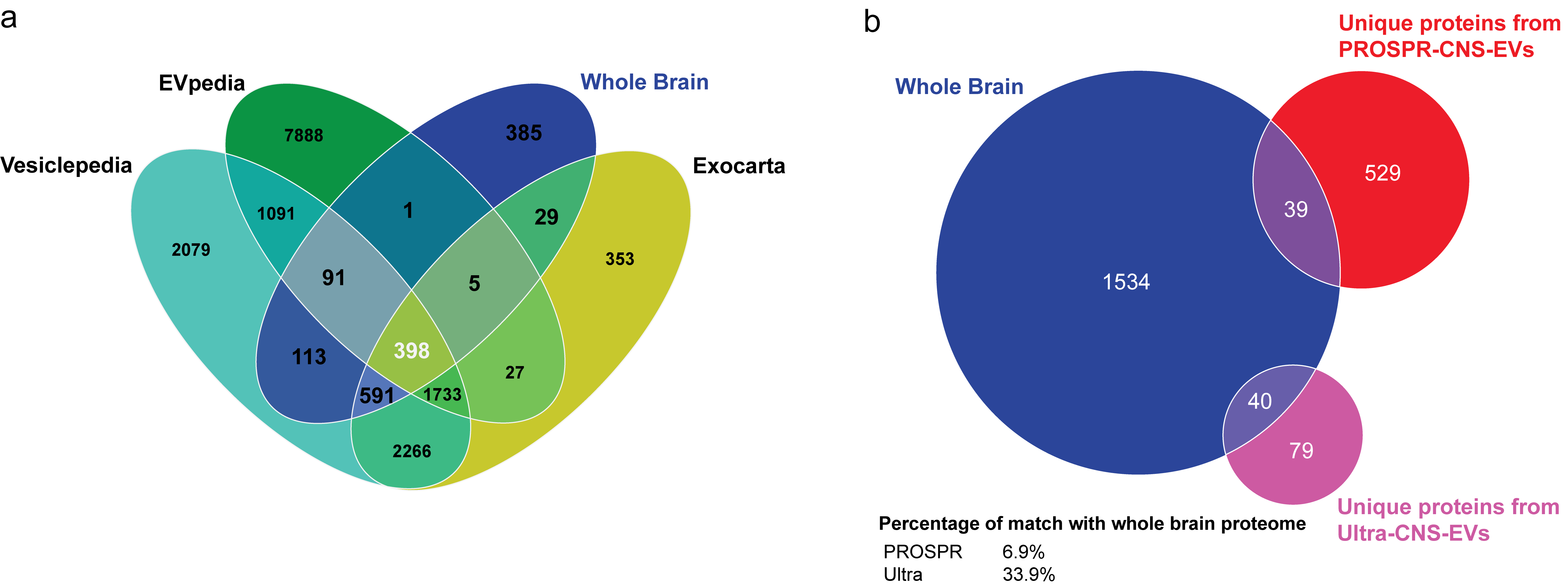

Supplement: Additional file 5: Figure S1. — Characterization of whole brain proteome. a. Venn diagram analysis of whole brain proteome matched to records in Exocarta [19], Vesiclepedia [21] and EVpedia [22] databases. b. Venn diagram analysis of whole brain proteome matched to the 568 unique proteins obtained from the comparison between PROSPR-CNS-EVs versus proteins compiled in Exocarta [19] and Vesiclepedia [21] and to the 119 unique proteins obtained in common with Ultra-CNS-EVs fractions (see in Fig. 2d the origin of these unique CNS-EV proteins). (TIF 1726 kb) [file 13024_2016_108_MOESM5_ESM.tif]
